# On Characterizing the Interactions between Proteins and Guanine Quadruplex Structures of Nucleic Acids

**DOI:** 10.1155/2017/9675348

**Published:** 2017-11-09

**Authors:** Ewan K. S. McRae, Evan P. Booy, Gay Pauline Padilla-Meier, Sean A. McKenna

**Affiliations:** ^1^Department of Chemistry, University of Manitoba, Winnipeg, MB, Canada; ^2^Department of Biochemistry & Medical Genetics, University of Manitoba, Winnipeg, MB, Canada; ^3^Manitoba Institute for Materials, University of Manitoba, Winnipeg, MB, Canada

## Abstract

Guanine quadruplexes (G4s) are four-stranded secondary structures of nucleic acids which are stabilized by noncanonical hydrogen bonding systems between the nitrogenous bases as well as extensive base stacking, or pi-pi, interactions. Formation of these structures in either genomic DNA or cellular RNA has the potential to affect cell biology in many facets including telomere maintenance, transcription, alternate splicing, and translation. Consequently, G4s have become therapeutic targets and several small molecule compounds have been developed which can bind such structures, yet little is known about how G4s interact with their native protein binding partners. This review focuses on the recognition of G4s by proteins and small peptides, comparing the modes of recognition that have thus far been observed. Emphasis will be placed on the information that has been gained through high-resolution crystallographic and NMR structures of G4/peptide complexes as well as biochemical investigations of binding specificity. By understanding the molecular features that lead to specificity of G4 binding by native proteins, we will be better equipped to target protein/G4 interactions for therapeutic purposes.

## 1. Introduction

Nucleic acid polymers that are rich in guanines have the potential to form a four-stranded structure called a guanine quadruplex (G4). When four guanines are arranged in a plane they are stabilized by hydrogen bonds between the Watson-Crick and Hoogsteen faces of adjacent guanines. These guanine tetrads have extensive surface area for pi-pi base stacking with other tetrads which contributes greatly to the thermodynamic stability of the G4 structure. All guanine G4s share this core feature of stacked tetrads of guanines, which is likely a common feature contributing to the recognition of G4s by proteins. Formation of G4 is dependent upon the presence of monovalent cations that relieve negative electrostatic charge repulsion from the O6 of guanines concentrated in the center of the tetrad. Positioning of monovalent cations (typically sodium or potassium) in the center of the G4, between two stacked tetrads, relieves the repulsive effect of the negative electrostatic potential [1, 2].

**Figure 1:**
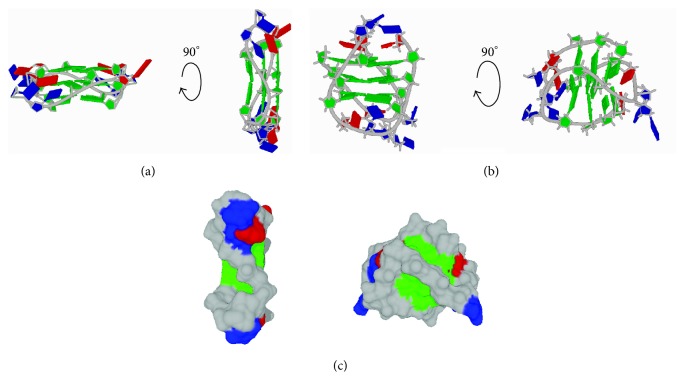
Side view of the human telomere guanine quadruplex in parallel (a) and antiparallel (b) conformations. Space filling models (c) for parallel (left) and antiparallel (right) quadruplexes. PDBs 1KF1 (a) and 143D (b). Guanines shown in green, thymines shown in blue, adenines shown in red, and the phosphoribose backbone shown in grey.

G4s can have greatly different topology based on the relative orientation of the phosphodiester backbone connecting the runs of guanines. If all the strands are parallel with respect to the 5′ and 3′ orientation of the ribose sugars, the loop sections between the runs of guanine must connect from the top to the bottom of the G4 in what is known as a propeller orientation (Figure 1(a)). The glycosidic bond angles in parallel G4s are typically all in the *anti conformation*, making this the preferred strand orientation for RNA G4s as it better accommodates the 2′ hydroxyl group. Similarly, locked nucleic acids (LNA) that use 2′-O,4′-C methylene linkages to force a C3′-*endo* sugar pucker will also strongly favor the all* anti *glycosidic bond conformation afforded by a parallel G4s [3]. For this reason, LNA have been utilized when forcing a parallel G4 topology is desired. For antiparallel strands the loops are referred to as lateral if connecting adjacent strands and diagonal if connecting strands opposite them; these create a mixture of* syn *and* anti *glycosidic bond angles and are the structures most often observed in DNA G4s [4] (Figure 1(b)).

Much like other nucleic acid secondary structures, distinct grooves created by the sugar-phosphate backbone can be observed in the space filling models of G4 structures (Figure 1(c)). These grooves as well as the large surface of the tetrads on either end of the G4 and the loops themselves represent the three feasible interaction sites for protein binding partners. Lateral and diagonal loops in antiparallel G4 leave the grooves accessible but would restrict access to the tetrad face. Propeller-like loops as seen in the parallel G4 pass directly over the grooves, allowing for intramolecular interactions of loop bases with the grooves, thereby obscuring the grooves but leaving the tetrad face more exposed for protein or ligand binding (Figure 1). Amongst the myriad of small molecule G4-binding ligands that have been synthesized one can find examples of ligands binding grooves, loop sequences, and tetrad faces of G4s. In some cases, binding of these ligands has been shown to stabilize, destabilize, and even alter the conformation of the G4 [5–7]. It would not be unreasonable to hypothesize that as our knowledge of protein-G4 interactions increases we will have examples of proteins that do the same.

Bioinformatic analyses of the human genome identified some ~350,000 unique, putative, G4 forming sites (PQS) [8] and high-throughput sequencing methods have identified a further 450,000 [9]. Interestingly, there appears to be an evolutionary pressure against PQS in protein coding regions and an enrichment of PQS in other noncoding regions when compared to pseudo-randomly generated DNA sequences. This enrichment of PQS in noncoding regions supports the idea that G4s may be key regulatory elements. Moreover, there is a statistical enrichment of PQS in oncogene promoter regions, some of which have been confirmed to exist in vivo by G4 specific cross-linking studies [10] and other biochemical investigations [11–14]. It has also become clear over the past decade that G4s play an important role in maintenance of telomere length [15–17] and ribosome biogenesis [18], which are disregulated in ~80% of cancers [19]. Furthermore, the expansion repeats of guanine rich regions in mRNA have been linked to two common forms of neurological disorders, Fragile X mental retardation [20], and familial Amyotrophic Lateral Sclerosis (ALS) [21].

There exists some controversy as to whether G4 can form* in vivo *and while significant effort has gone towards testing this question experimentally, observing the structure of nucleic acids* in vivo *is a monumental task that is prone to biasing dependent on the method used to determine structure. One method that is used to visualize unique motifs in cells is immunofluorescence. The Balasubramanian group has developed antibodies to selectively target both DNA [22, 23] and RNA G4 [24] and successfully used them to visualize these structures* in vivo*. Furthermore, one could reason that the physiological effects of quadruplex-specific small molecules on cells are evidence of quadruplex formation* in vivo. *In these cases, the arguments can be made that the antibody or small molecule is merely inducing the formation of G4 and that the structures do not exist in the absence of these probes or that the specificity of the antibodies/drugs has changed in a cellular context and that they are no longer binding to G4 but exerting an effect through interactions with other structures. A recent study using high-throughput sequencing and* in vivo* chemical modifications has indicated that RNA G4s are globally unfolded in some mammalian cell lines [25]. A similar critique can be made that the chemicals added for modification of nucleic acids could be altering their structure* in vivo* or that the concentration of chemical reagent added results in nonspecific effects. Regardless of being able to prove the existence of G4s* in vivo,* many proteins have highly conserved G4 specific binding domains that would likely not have persisted in eukaryotes if they had no biological function. One way to probe this purpose is by disruption of the protein-G4 interaction.

Targeting G4-protein interactions with small molecule drugs has potential to treat a multitude of diseases [17, 26, 27]. Currently there is one such compound in clinical trials for cancer treatment, that is, CX-3543 or Quarfloxin. While originally selected for its interaction with the MYC G4, CX-3543 has been shown to localize to cell nucleoli where it inhibits Pol I transcription by blocking the interaction between nucleolin and G4s in rDNA [18]. As well as being targets of small molecule therapeutics, G4s themselves have been employed as therapeutics to target specific proteins. One such example is thrombin binding DNA aptamers that inhibit thrombin-induced platelet aggregation and clot-bound thrombin; these have potential for use as anticoagulants during various surgeries and are currently in clinical trials [28]. Although thrombin is not by nature a G4-binding protein, G4 aptamers display remarkable affinity and specificity for regions of thrombin that normally interact with other proteins.

To better understand the mechanism of G4-mediated cellular processes and how small molecules interfere with them, it is imperative to understand how proteins recognize their endogenous G4 binding partners. While many G4-binding proteins have been identified and validated [29], there is relatively little information on how they specifically recognize G4s. Typical approaches to study this include altering some of the features of either the G4 substrate or protein, subsequently assessing how these mutations affect the interaction, and inferring from this their role in G4 recognition. This method is relatively simple and inexpensive to perform and can yield useful information; however it is not always trivial to show that such mutations are directly mediating the interaction and not indirectly affecting the interaction by altering the conformation of the mutated species. Small-angle X-ray scattering is a solution-based technique that can be used to determine low-resolution models of protein and nucleic acid. Though it does not provide enough resolution to distinguish individual atoms, it can provide insight into the shape and orientation of species in a protein nucleic acid complex that can be complementary to other data obtained from mutation analysis and/or other higher-resolution structural methods such as NMR and X-ray crystallography. While there are currently over 250 high-resolution NMR and X-ray structures of G4s deposited on the Research Collaboratory for Structural Bioinformatics (RCSB) website and nucleic acid database (NDB), comparatively few high-resolution structures of protein interacting with G4 have been solved. The most extensively studied interaction is between G4 containing DNA aptamers and thrombin, on which a number of structures have been deposited (PDB: 1HAP, 1HAO, 3QLP, 4DII, 4DIH, 5CMX, 4I7Y, 4LZ1, 4LZ4, 5LUW, 5LUY, 5EW1, and 5EW2). More recently the structure of a peptide from the prion protein bound by an RNA G4 aptamer has been solved (2RSK, 2RU7). Currently, to our knowledge, only 3 high-resolution structures have been solved showing G4-peptide interactions involving proteins that interact with G4 as part of their normal cellular function (2LA5, 5DE5, and 2N21). Herein we will discuss the insights gained from these structures as well as some key mutational analysis studies and look at the benefits, as well as drawbacks, of these approaches.

## 2. Thrombin Binding by G4 DNA Aptamers

The development of thrombin binding DNA aptamers for their anticoagulant properties has been ongoing since the early 1990s; although thrombin is not naturally a G4-binding protein, the 15-base DNA G4 (TBA), developed through SELEX, can tightly bind thrombin and inhibit fibrin clot formation [31, 32]. This nucleic acid sequence forms an antiparallel G4 with two guanine tetrads connected by three lateral loops: one face of the G4 is covered by two TT loops and the other by a TTA loop. Initial high-resolution structures of TBA (1HAP, 1HAO) showed that the fibrinogen binding site of thrombin (exosite I) was likely interacting with the loops of TBA; however, due to lack of electron density in the loop regions it was unclear whether this interaction was with the TT loops or TTA loop [33]. 15 years later 3 structures (3QLP, 4DII, and 4DIH) were produced from the lab of Filomena Sica that definitively show the TT loops acting as a pincer that bind either side of the protruding region of exosite I utilizing multiple polar and hydrophobic contacts [34, 35].

**Figure 2:**
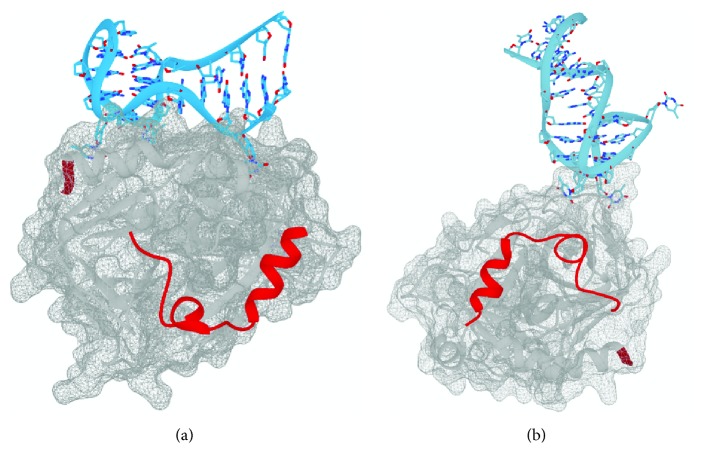
X-ray structures of HD22-27mer (a) and RE31 (b) guanine quadruplex-containing aptamers bound to thrombin, for ease of differentiating the thrombin binding sites: shown in red ribbon is the light chain of thrombin and shown in grey ribbon is the heavy chain of thrombin with the C-terminal nub of the heavy chain shown in red. DNA aptamers are shown in blue. PDBs 4I7Y (a) and 5CMX (b).

Many variants of the aptamer have been made that enhance the stability of, affinity for, and inhibition of thrombin. One such potent second-generation thrombin aptamer is the HD22-27mer [36] for which a high-resolution X-ray structure (4IY7) of the aptamer-thrombin complex has been solved [37] (Figure 2(a)). HD22-27mer forms a mixed duplex-G4 structure where the duplex is directly enchained to the G4. The HD22-27mer forms an unusual pseudo-G4 topology with four loops, where the first loop connects two guanines in the same tetrad and the remaining loops form a more typical lateral antiparallel loop pattern. The 2nd and 4th of these loops interact with each other through Watson-Crick A-T hydrogen bonds and cap one side of the G4, while the intratetrad loop and the 3rd loop also form A-T hydrogen bonds and cap the opposite side of the G4. In contrast to the structure of thrombin and TBA, protein recognition of HD22-27mer involves the extended double stranded region as well as a bulged-out thymine and the pseudo-G4 core and appears to involve exosite II and not exosite I. G20 is the last guanine before the duplex region and forms three polar contacts with Arginine 93, two through ribose hydroxyl groups and one through its phosphate group. Loops 2 and 4 of the G4 contribute to the stability of the complex by forming multiple hydrophobic interactions with thrombin but, unlike with TBA, only thymine 9 has multiple polar contacts with exosite II.

Recently a 31-nucleotide third-generation thrombin aptamer, RE31, was crystalized in complex with thrombin [38] (Figure 2(b)). Like the HD22-27mer, RE31 has an extended duplex region as well as a two-tetrad G4. Unlike HD22-27mer the duplex region of RE31 exhibits continuous base stacking with the G4 rather and the duplex region does not appear to be important for binding to thrombin. Despite the presence of a G4-duplex junction, the binding of RE31 to thrombin is much more similar to TBA than HD22-27mer; both molecules use the TT loops to interact with exosite I through hydrogen bonds and hydrophobic interactions.

Despite the persistence of a two-tetrad G4 in these thrombin binding aptamers, interactions with the G4 core are not observed in any of the structures. Instead, the main site of interaction, which is common between all the structures mentioned above, is with the second and fourth loop of the G4. While TBA and RE31 use mainly polar contacts and some ancillary hydrophobic interactions to interact with exosite I of thrombin, the G4 loops of HD22-27mer use mostly hydrophobic interactions to bind exosite II. This common mechanism of binding to different sites of thrombin suggests that the G4 acts as a scaffold that presents the single stranded loops to the binding pocket on the protein.

## 3. Interaction between the RGG Motif and G4 Structures

Motifs rich in arginine and glycine have been known to play functional roles in numerous physiological processes, usually involving nucleic acid interactions, for many decades [39]. Glycine is the most flexible of the amino acid side chains in terms of its accessible dihedral angles; thus sequences rich in glycine can sample a large variety of conformations allowing them to be very promiscuous binding partners. Arginine is a positively charged amino acid with delocalized pi electrons. This allows for favorable electrostatic interactions with the phosphate backbone of nucleic acids, as well as potential base stacking with the nitrogenous bases and hydrogen bonding in as many as three different directions via its guanidinium moiety. Many of the currently known G4-binding proteins have RGG/RG motifs [29, 39], some of which have been shown to be critical to G4 binding and recognition, including the translated in sarcoma/fused in sarcoma (TLS/FUS) [40], nucleolin (NCL) [41], Ewing's sarcoma protein (EWS) [42], Epstein Barr virus nuclear antigen 1 EBNA1 [43], DDX21 [44], and fragile X mental retardation protein (FMRP) [45].

Since the most obvious structurally unique feature of a G4 is the tetrad face, one might expect the arginine of the RGG motifs to contribute to base stacking interactions with the terminal guanine tetrads. However, there is only indirect evidence that the tetrad face plays a role in RGG mediated interactions. Many small molecule G4-binding ligands have been shown to interact tightly with the tetrad face via pi-pi stacking interactions and two of such molecules, BRACO-19 and CX-3543, have been shown to disrupt interactions with G4-binding proteins (EBNA1) [43] and NCL [18], which contain the RGG motif. While this indirectly implicates competition with the protein for the tetrad face of the G4, these compounds also bind electrostatically to the grooves and loops of the G4 [46, 47] possibly preventing protein-groove interactions or reorienting the loop structure to disrupt protein binding in such a manner.

### 3.1. Recognition of Phosphoribose Backbone of G4 Loops by RGG/RG Domains

Biochemical investigations in the lab of Takanori Oyoshi have revealed that the RGG domains of the EWS and FUS/TLS proteins are critical for G4 binding and do so through interactions with the phosphoribose backbone of the loop regions [48–50]. The EWS protein's RGG3 domain has high affinity for specific RNA and DNA G4 that is dependent on the arginines in this region [42]. Interestingly this binding seems to be dependent on the presence of loops of at least 2 nucleotides and higher affinity is observed with longer loops [50]. Replacing the loop sequences with abasic deoxy-ribose backbones did not affect this affinity, indicating that the interaction was with the backbone itself and not the nucleotide bases. In contrast, both the backbone and bases of the TT loops in TBAs are involved in thrombin binding and thus replacing the TT loop residues of TBAs with abasic backbone reduces the affinity for thrombin as well as the orientation of the aptamer on the surface of thrombin [51–53]. Furthermore, an intermolecular G4 with single stranded overhangs did not demonstrate the same affinity, indicating the structure of the backbone in the G4 loops was important for recognition by the RGG3 domain. While the initial strand orientation of the G4 did not affect binding, RGG3 was shown by circular dichroism to convert the topology of DNA G4 from antiparallel to parallel. Thus, it seems that the phosphoribose backbone structure in the propeller type loop conformation is being specifically recognized by the RGG3 domain of EWS [50].

Mutation of the three phenylalanine residues in the RGG domain of FUS/TLS to tyrosine eliminates the binding to the DNA G4 while retaining affinity for the RNA G4 [48]. Interestingly, when the DNA G4 loops were replaced with an abasic phosphoribose backbone, the binding capability was restored. This indicates that the tyrosine residue specifically recognizes the 2′ hydroxyl group of ribose sugars in the loop region and not the guanine tetrads. RNA G4 with no loops or locked nucleic acid loops (which obscure the 2′ hydroxyl) abrogate the binding, consistent with the notion of 2′ hydroxyl recognition being important and ruling out possible differences caused by strand orientation in the DNA G4. Truncation of the protein to exclude two tyrosine residues in the native RGG domain of TLS/FUS abolishes the affinity for RNA, while retaining the affinity for DNA, G4 [48]. Truncation of the section containing three phenylalanines has the opposite effect, maintaining affinity for RNA, while losing affinity for DNA, G4. Mutations of these aromatic residues to alanine abolish all affinity for G4, suggesting that their interactions are not only important for specificity to the ribose sugar but also critical for G4 recognition [49].

These mutational studies with EWS and FUS/TLS demonstrate that RGG domains, like in the case of thrombin binding aptamers, can recognize the loops of G4 structures. Both RGG domains preferentially interact with G4-containing longer loops as opposed to those lacking loops. Furthermore, the RGG domains of EWS and FUS/TLS need only the phosphoribose backbone to be present in the loops for full affinity, whereas thrombin interacts with both the backbone and bases of TBA loops. This indicates that the binding of G4 by EWS and FUS/TLS is not sequence specific and that the position of the backbone of the loops is likely key to specific recognition by EWS and FUS/TLS.

### 3.2. G4 Binding by the RGGGGR Peptide of the FMRP Protein

**Figure 3:**
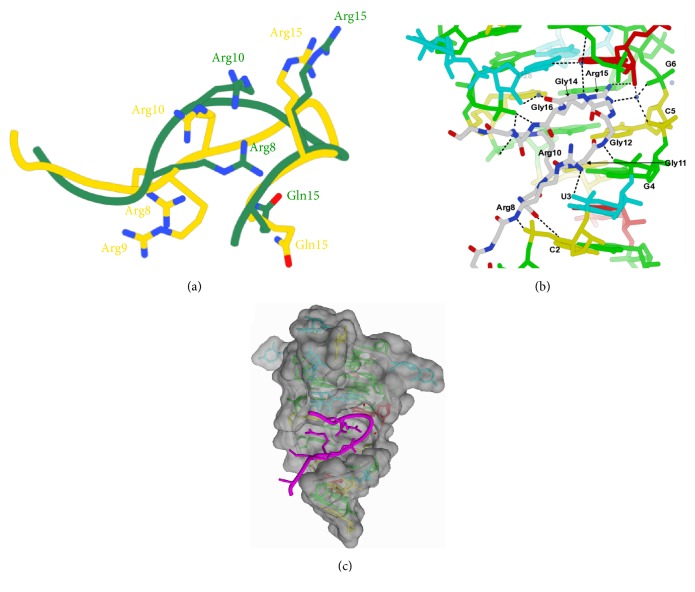
RGGGGR peptide bound to SC1 RNA quadruplex-duplex junction. Comparison of peptide conformations between X-ray (green) and NMR (yellow) structures shown in (a). Hydrogen bonding pattern between peptide and nucleic acids shown in (b). Space filling model shown in (c), with peptide shown as a magenta ribbon. PDBs 5DEA (X-ray) and 2LA5 (NMR).

Currently the only high-resolution structures of an RGG motif binding to G4 are both from the laboratory of Dinshaw Patel (2LA5, 5DE5) where they have studied the interaction between the 26-amino-acid RGG box peptide of FMRP and 36 nucleotides of the SC1 (transcription factor 19) RNA, which forms a mixed G4-duplex structure, by NMR [54] and X-ray crystallography [55] (Figure 3). This short peptide has been previously shown to retain specificity and affinity for G4 structures and not other RNA that the full-length FMRP protein interacts with, indicating it is responsible for G4 recognition by FMRP [56]. Discrepancies between the structures are attributed to flexible regions within the peptide and loops of the RNA (Figure 3(a)). The structure shows a G4 of parallel strand orientation with 3 guanine tetrads and an unusual mixed base tetrad (GUAU) before the duplex junction. While there are no direct interactions with the guanine tetrads, Arg15, which was shown to be indispensable for binding, is seen to exhibit a cation-pi stacking interaction with A17, which is part of the mixed base tetrad. In addition to this stacking interaction, Arg15 is observed in a hydrogen bond with the Hoogsteen face of G7, the first base in the double stranded region (Figure 3(b)). Arg10, another indispensable residue, rests in between the first and second bases of the duplex region (C30 and G31) and exhibits a cation-pi interaction with C30 and forms multiple hydrogen bonds with G31 and the phosphoribose backbone. Mutation of these first two base pairs has been shown to reduce the affinity of the peptide for SC1 tenfold [56]. The two arginines that make contact with these bases act as an anchor at the base of the G4 stabilizing the junction from four- to two-stranded RNA, a feature that was exemplified by RNase digestion experiments.

The four glycines between Arg10 and Arg15 form a tight beta-turn motif and they make many important contacts with the RNA. Gly11 appears to be indispensable (based on mutations made to the peptide), as it coordinates hydrogen bonds from its backbone NH and CO to the carbonyl of G4 and the NH_2_ group of C5 in the duplex region. Gly14 makes the second contact with the G4 region via a hydrogen bond from its backbone amide to the ribose sugar of the uridine in the mixed tetrad (Figure 3(b)).

Unlike the thrombin binding aptamers and RGG domains of EWS and FUS/TLS, the SC1 RNA-FMRP interactions do not involve the loop bases at all; the high-resolution structures and mutational analysis show that the important interactions are with the duplex region adjacent to the G4 as well as the mixed base tetrad. From the space filling model (Figure 3(c)) it appears that the RGGGGR peptide is occupying a groove created by the tetraplex to duplex junction; thus its mode of recognition appears to be a mixture of van der Waals space filling and sequence specific interactions mediated by the placement of arginines in this cavity.

## 4. G4 Recognition of the Tetrad Face by the DHX36 Specific Motif

**Figure 4:**
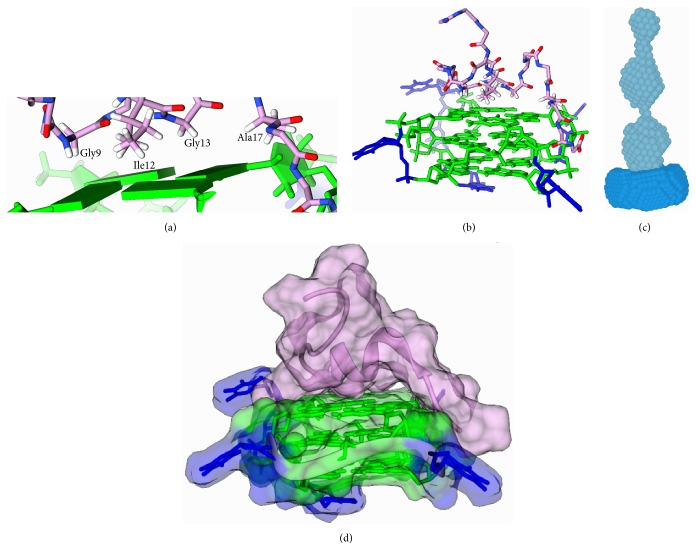
NMR structure of 16-amino-acid peptide (pink) from DHX36 bound to a DNA guanine quadruplex (green and blue). Amino acids that stack on the face of the guanine tetrad are labelled in (a) and charged amino acids that interact with the backbone of the quadruplex are shown in (b). Small-angle X-ray scattering data of a 52-amino-acid fragment of DHX36 (turquoise) bound to an RNA quadruplex (blue) shown in (c). Transparent space filling diagram of the 16-amino-acid DHX36 peptide bound to DNA quadruplex. PDB 2N21 (NMR) and SAX structure [30].

The protein DHX36 is a helicase enzyme that has the capability to bind and unwind RNA G4s with great specificity [57, 58]. A region of 13 amino acids that is unique to DHX36 is evolutionarily conserved amongst higher eukaryotes and is both necessary and sufficient for binding of G4s [59]. Low-resolution small-angle X-ray scattering has shown that the G4 recognition motif is oriented towards the tetrad face (Figure 4(c)) [30, 60] and mutations to the loop sequence of an endogenous G4 binding partner resulted in a reduced affinity, indicating that loop conformation may also be important for recognition of G4 by this motif [61]. A high-resolution NMR structure from the lab of Anh Tuân Phan showing a small 18-amino-acid peptide bound to a synthetic DNA G4 TT(GGGT)x4 (2N21) supports this mode of binding [62]. It is worth noting that a similar mode of binding has been observed between an RNA aptamer and a 16-amino-acid peptide from the N-terminus of the prion protein PrP^C^ (2RSK and 2RU7) [63, 64].

Since DHX36 has significant preference for the parallel versus antiparallel loop orientation, the loop sequences were limited to a single thymine residue which promotes formation of the parallel species. The structure shows the propeller type loop arrangement typical of parallel G4s with the peptide forming a short alpha-helical region followed by a turn that covers the entire tetrad face. The two aromatic residues (Trp13 and Tyr14) do not make any contacts with the DNA but are purported to stabilize the L shape of the peptide. Four residues, namely, Gly9, Gly13, Ile12, and Ala17, are found at the tetrad-peptide interface close enough to permit CH-pi interactions (Figure 4(a)).

Positively charged residues Lys8, Arg10, and Lys19 are each in close enough proximity to the grooves of the G4 to form electrostatic interactions which could help stabilize the observed interaction (Figures 4(b)/4(d)); however, previous mutation studies have shown Lys8 and Arg10 to be dispensable in the context of the full-length protein [59]. The observed mode of binding explains DHX36's preference for RNA G4s; since RNA G4s form almost exclusively the parallel strand orientation with propeller type loops, they are expected to have exposed tetrad faces. Binding to a large exposed hydrophobic surface like the tetrad face provides a significant increase in entropy through desolvation and is thus a highly favorable interaction. DNA G4s which have antiparallel or hybrid type loop structures can avoid this entropy penalty through interactions between the loop bases and tetrad face which bring the polar sugar-phosphate backbone over the top of the G4 and shield the hydrophobic tetrad face.

*Limitations of NMR and X-Ray Crystallography*. NMR and X-ray crystallography are two techniques that have many strengths and limitations which complement each other and make the use of both techniques the ideal method to investigate biological structures of interest. Where X-ray crystallography will provide a single image of the exact structure in the crystal lattice, NMR will show many models of possible structures which all fit the data equally well. NMR is a solution-based technique which can view the dynamics of molecules on a physiological time scale, whereas X-ray crystallography views, usually, the most thermodynamically stable form of a system. When analyzing X-ray and NMR structures, it is important to remember that they provide only a snapshot of a dynamic process, usually under nonphysiological conditions. Insights gleamed from these snapshots should be tested with mutational studies that use the information from the structure to test its validity.

The main pitfall of NMR for structural biology is the size restriction on the molecule being investigated. Especially for proteins and nucleic acids, past a certain length, there are many overlapping signals from different regions of the biomolecule resulting in broad, indiscrete peaks, which makes separation and identification of which signal came from which atom an immense challenge. Therefore, often, only small peptides are examined by this method. The disadvantage of this approach is that you cannot ensure that the small peptide is not folded as it would be in the context of the full-length protein. For example, the 18-amino-acid peptide from DHX36 is only 2% of the size of the full 1008-amino-acid protein. While the peptide does demonstrate significant affinity for quadruplex, the full-length protein binds much more tightly [59]. Thus any structural insights gained from examining the peptide are, at best, only a piece of the full picture. Furthermore, it has been observed from the structures of the FMRP/Sc1 complex that recognition of G4s is not just from the G4 itself but the junction regions and adjacent double or single stranded regions; thus it is also preferable to use a larger oligonucleotide which better mimics biological RNA/DNA species. The problem with doing so is that such complexes can exceed the size limit for structural determinations by NMR and that such large complexes are usually difficult to crystallize, often requiring nonnative salt conditions or stabilizing antibodies. The disadvantage to such crystallization approaches is the difficulty to prove reliability of any single structure obtained in terms of physiological relevance.

One of the advantages of NMR approaches is the ability to identify disordered regions. Even without relaxation dispersion experiments, the variability in models obtained by NMR can hint at which regions are disordered. X-ray crystallography, on the other hand, traps disordered regions into a single stable conformation which can confuse data interpretation and cause misleading results. An example of this trapping of flexible regions is in the X-ray structure of the FMRP RGG domain and the Sc1 RNA. The NMR data on the complex, which was obtained first, had poorly defined loop regions, hinting at conformational flexibility. Fortuitously two different loop conformations were captured in a single asymmetric unit in the crystal conformation supporting the notion that the loop region samples multiple conformations. Had there been only one complex in the unit cell, there would be less certainty in the dynamics of the loop nucleotides.

Another consideration for studying G4s by either NMR or crystallography techniques is that they require high biomolecule concentrations that can lead to artifacts being observed in both the crystal lattice and the NMR data. For example, the first structure of a natural G4 and protein in the same crystal unit cell was observed for the* Oxytrichia nova* telomere end binding protein (OnTEBP). OnTEBP has been shown to bind to the single stranded repeats at the end of telomeres (TTTTGGGG in* Oxytrichia nova*) and protect them from DNA damage repair mechanisms which recognize breaks in dsDNA [65]. Horvath and Schultz [66] were studying the binding of the single stranded repeat (GGGGTTTTGGGG) to the OnTEBP region and upon analyzing their X-ray crystallography data observed diffraction from a DNA quadruplex which appeared to be bound to OnTEBP (1JB7). Three symmetry related OnTEBP proteins appeared in the unit cell each bound to the ssDNA and each contributing to the binding of a single DNA quadruplex formed from a dimer of their single stranded substrate. Interactions with one OnTEBP protein were with a major groove in the G4 backbone and featured both electrostatic and hydrogen bonds to the deoxyribose sugar groups. The other two OnTEBP proteins bound via the TTTT loop sequences via electrostatic interactions, hydrogen bonds, and pi-pi as well as CH-pi stacking interactions. The authors recognized in their paper that the interactions were likely due to crystal packing interactions and may not be physiologically relevant, which serves as an example of why one must take care when analyzing data which was acquired under nonphysiological conditions.

Conditions such as low pH, high salt, and high sample concentration are often used to obtain either better diffracting crystals or more discrete NMR bands. Figure 1 exemplifies the importance of careful consideration of the solution in which structural information is obtained as well as the method of obtaining such information. Two completely different topologies are observed for the same DNA sequence dependent upon which cation is stabilizing the quadruplex. The presence of Na^+^ stabilizes the antiparallel [67, 68] form whereas K^+^ enables crystallization of the parallel [69] form.

## 5. Conclusions

Mutational analysis and high-resolution NMR and X-ray studies have provided invaluable insights into the structures of biological molecules; this in turn has expounded the molecular mechanism of many important biological processes. With these structure function relationships, we can rationally approach how to alter these processes and modify them to fix pathologies. Although the recognition of G4s by proteins is far from being understood, several trends have emerged amongst the data collected to date. It is apparent that the loop structure of G4s and the junction regions adjacent to them present a unique molecular landscape for recognition by proteins. So far this appears to be the most common mode of recognition. Thrombin binding aptamers appear to canonically bind thrombin using the thymine bases of two lateral loops like a pincer to latch onto thrombin. The RGG domains of EWS and FUS/TLS also appear to interact mainly with the loops of G4s; the mode of recognition in this case is likely from the placement of the phosphoribose backbone that connects the strands of the G4 since the bases themselves have been shown to be dispensable. In the case of FMRP, the RGGGGR peptide stabilizes the transition from G4 to duplex by filling the junction between them with base stacking and Hoogsteen type hydrogen bonds with the double stranded region.

Mutations made to the FUS/TLS protein and its G4 substrate have shown that preferential recognition of RNA over DNA G4 can be mediated by 2′OH interactions. It will be interesting to see how other G4-binding proteins are affected by such mutations as those performed with FUS/TLS protein. So far, DHX36 is the only G4-binding protein that has been shown to directly interact with the open guanine-tetrad face of the G4 and this satisfactorily explains its preference for RNA G4s, since the faces of antiparallel G4s are typically obscured by the loops. However, since much of the affinity for G4 is lost when only examining the DHX36 specific motif peptide, there are likely other binding interfaces on the G4 which contribute to the full-length protein's binding affinity.

Currently most G4-binding small molecules interact directly with the guanine-tetrad face. This would, obviously, directly compete with DHX36 binding but has also been shown to prevent binding of other RGG domain containing G4-binding proteins, making specific targeting and mechanistic elucidation of their effects challenging [18, 43, 47]. An alternate approach to designing compounds to bind G4s would be to design compounds that mimic their unique loop structure. Since loop recognition seems to be key to the specificity of many G4-binding proteins, creating small molecules to compete with this interaction may result in more specific compounds that could be used in a more targeted manner to affect cellular pathways.
